# Annotations

**Published:** 1908-01-25

**Authors:** 


### ANNOTATIONS.

### Singing in Relation to Pulmonary Disease.

In the current number of the *British Journal of Tuberculosis* Dr. Murray Leslie and Dr. Cyril Horsford direct attention to the value of singing, and of the vocal and respiratory exercises associated with the cultivation of that art, in the prevention and cure of diseases of the lungs. They consider that increased professional recognition should be extended to this special therapeutic agency, and they contend that it may be advised in (1) persons in whom, either from family predisposition or from individual weakness or abnormality of the chest, the onset of pulmonary consumption is to be feared; (2) early cases of consumption as soon as the disease becomes quiescent; (3) certain more advanced cases where no active disease or ulceration is in progress. To secure the desired end it is suggested that some public institution should be founded, either independently or as a special department of our already existing Colleges of Singing, and that such institution should be open to suitable cases referred from the hospitals. The beneficial influence of singing is exerted in several different directions. First it involves correct nasal breathing, and this means that the air admitted to the lungs is practically germ-free, and also the adequate development of the upper portions of the respiratory passages. A second effect is seen in the maintenance of the elasticity and proper expansion of the chest. The necessary breathing exercises mean increased functional activity of all parts of the lungs, including the apices, where, as is well known, tuberculosis commonly commences—a fact which is doubtless due, at least in part, to the limited expansion which occurs in these regions in ordinary circumstances. Lastly may be mentioned the improved oxygenation of the blood, which singing and efficient pulmonary respiration necessarily promote. The suggestion that singing may be used in the fight against pulmonary tuberculosis is an interesting one, and is a further instance of the therapeutic value of hygienic measures, which is so large an item in the current professorial creed.

### Observations on the Knee-jerk.

The condition of the knee-jerks is a commonplace of the ordinary routine clinical examination of the individual patient, and, at least in general terms, the significance of any decided departure from the normal is well understood. Yet there are one or two points in connection with this sign upon which more confident information is much to be desired. Perhaps the most important is the question whether absence of the jerk necessarily means organic disease, using that term to include changes in the peripheral nerves as well as in the spinal cord. No doubt when such absence coincides with other suggestions of anatomical change it is universally accepted as confirmatory evidence of the highest order. But a difficulty arises when the defect exists as the one and only peculiarity of the nervous system, as this is tested by clinical examination. Such a state of matters is not common; still it occurs occasionally, and when noted, a sure interpretation is not readily provided. By some authorities the absence is declared to be definitely pathological, while by others it is regarded as a possible physiological peculiarity. Again, the criticism is sometimes made that in some cases of reputed absence of the jerk the conclusion depends on inadequate methods of examination. Allowing this to be occasionally possible, it still remains true that there are well-sustained instances in which such absence is beyond question, and where, also, the nervous system is otherwise free from suspicion. What, however, is not so certain is the earlier or later fate of such patients. Is the absence permanent or temporary? May it be a congenital peculiarity or defect? Does evidence of such a disease as tabes dorsalis appear at a later date in any considerable proportion of these cases? These are questions to which definite answers are much to be desired, and for such answers reliance must be mainly placed on those who possess the opportunities offered by general practice.

### Professor Koch on Sleeping Sickness.

The statement made by Professor Koch in reference to the results of his investigations into the nature and treatment of sleeping sickness will be read with much interest. In the first place his observations, made on the spot, confirm the appalling mortality which is produced by this disease. In one part of Uganda the population was formerly 300,000 and is now reduced to 100,000; and about Victoria Nyanza, where Koch established his headquarters, "Natives dead or dying of the scourge were," he says, "to be found on all sides." Of the value of atoxyl in the treatment of the disease he expresses a very confident opinion, and he attributes adverse views to the observation of cases in which the remedy has been used at too late a stage. In the later developments of sleeping sickness a good result is not to be expected from atoxyl, but administered in the early stages decided benefit will be produced, and there may even be a complete cure. Regarding the extension of the disease Koch's researches carry the position a step further than the infection of human beings by the bite of a specific fly. He advances the suggestion that these flies feed especially, if not entirely, upon crocodiles, and crocodiles' blood may be found in their stomachs. Hence, it may be anticipated that with the destruction of these reptiles, the specific fly and the disease which this conveys will disappear. Professor Koch points out that it is a comparatively easy matter, as crocodiles deposit their eggs in well-known places, and with the destruction of the eggs will come the extinction of the species. The natives, it would appear, are more difficult to deal with. They have no enthusiasm for the new theory, and they oppose, at least passively, the segregation camps which are necessary for the isolation of infected individuals. It is satisfactory to know that Professor Koch disbelieves in the suggestion that sleeping sickness will extend to Rhodesia, and he points out that the specific fly by which the disease is conveyed does not exist in that country. This is the more satisfactory in view of the somewhat alarming statements which have recently been made on this point.

